# A rare case report of complications in craniofacial injuries: Cavernous sinus-carotid fistula

**DOI:** 10.1097/MD.0000000000009511

**Published:** 2017-12-29

**Authors:** Dong Wang, Xiao-Mei Sun, Jin Wu

**Affiliations:** a Department of Oral and Maxillofacial Surgery, Dongguan People's Hospital, Dongguan; b Department of Pediatrics, West China Second Hospital, Sichuan University; c Key Laboratory of Birth Defects and Related Diseases of Women and Children (Sichuan University), Ministry of Education, Chengdu, China.

**Keywords:** case report, CCF, craniofacial injuries, maxillofacial surgical care

## Abstract

**Rationale:**

Cavernous sinus-carotid fistula (CCF) is a rare complication of craniofacial injuries in patients receiving oral and maxillofacial surgical care.

**Patient concerns:**

A retrospective review of 15 patients with CCF records was conducted. In addition, we present a rare case of a 32-year-old Chinese woman with CCF receiving oral and maxillofacial surgical care.

**Diagnoses:**

Digital subtraction angiography (DSA) confirmed a diagnosis of CCF.

**Interventions:**

Endovascular surgery was performed via the femoral artery under general anesthesia without any complications.

**Outcomes:**

The postoperative course was uneventful, and the subjective and objective ophthalmic symptoms had resolved.

**Lessons:**

The symptoms of CCF may be delayed for several days or weeks. Thus, maxillofacial surgeons should be aware of this and avoid the untimely repair of facial fractures with potentially disastrous consequences. Interventional neuroradiologic approaches that involve the use of a detachable balloon have made it possible to care for patients with CCF without any complications.

## 1 Introduction

Cavernous sinus-carotid fistula (CCF) is a rare complication of craniofacial injuries, occurring in only 0.17% to 0.27% of cases, according to data reported in the literature.^[1–2]^ Recent advancements, including new imaging techniques and improved surgical methods, have improved the diagnosis and treatment of CCF. However, its diagnosis and treatment are still major challenges for oral maxillofacial surgeons, because of the low incidence of CCF. Furthermore, untreated CCF may cause serious permanent neurologic and visual deficits or fatal epistaxis. This paper describes 15 patients diagnosed with CCF from 2000 to 2015.

## 2 Presentation of cases

### 2.1 Clinical data

This paper describes 12 Chinese men and 3 Chinese women diagnosed with CCF. There was a strong prevalence of young male patients aged 19 to 40 years, whereas the majority of the older patients were women (Table 1). The causes were a traffic accident (5 patients), beating (5 patients), falling (2 patients), and undefined (1 patient). In addition, there were 2 female patients with spontaneous cavernous-carotid sinus fistula (SCCF) without an identified cause. 14 patients presented with CCF on either side (5 right and 9 left), and 1 patient presented with CCF on both sides. Two patients displayed symptoms immediately after injury, and 13 patients displayed delayed symptoms, with the longest delay being 2 months after injury. The clinical findings were intracranial bruit (10 cases), pulsating exophalmos (14 cases), conjunctival chemosis (15 cases), oculomotor palsy (1 case), decreased visual acuity (8 cases), headache and tinnitus (10 cases), intracranial hemorrhage and epistaxis (3 cases), diplopia (3 cases), and fundus oculi lesions (7 cases) (Table 2). Thirteen patients had cranio-maxillofacial fractures (3 cases on the temple, 3 cases on the frontal portion, 3 cases on the skull base, 1 case on the orbit, 1 case on the zygomatic bone, and 1 case on the Le Fort III of the maxilla), 1 patient had a mandible fracture. Four patients underwent common carotid artery (c. A), internal carotid artery (ICA), or external earofid c. A ligation. In addition, the ICA isolation method was used on 2 patients, and interventional therapy was used on another 2 patients. The results were satisfactory for all 8 patients. Another 7 patients did not undergo surgery and outcomes were incurable; only 1 patient displayed motor aphasia and center facial paralysis after left common c. A and ICA ligation. Written informed consent was obtained from all patients.

**Table 1:**
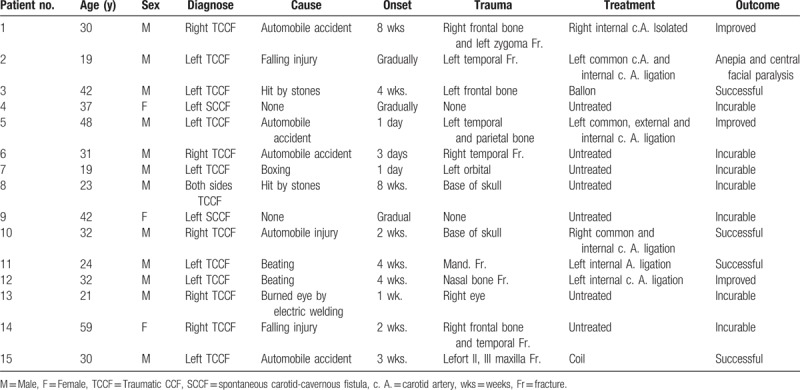
Treatment and outcome of 15 patients with cavernous sinus-carotid fistula.

**Table 2:**
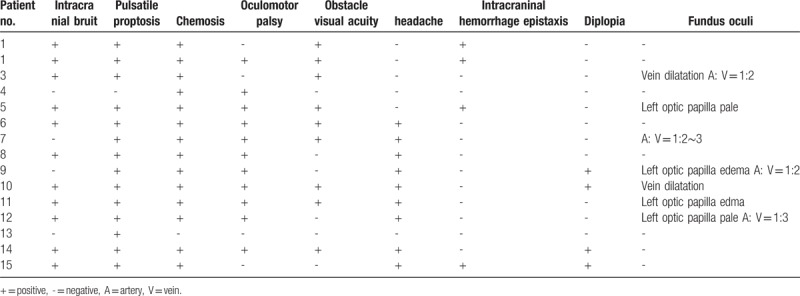
The clinical features of patients with cavernous sinus-carotid fistula.

### 2.2 Report of a rare case receiving oral and maxillofacial surgical care

A 32-year-old Chinese female receiving oral and maxillofacial surgical care was involved in a motor vehicle accident 1 week prior and sustained a closed facial injury. Although she lost consciousness for less than 1 hour, there was no evidence of retrograde amnesia. Computed tomography (CT) disclosed a bilateral zygomatic fracture. In addition, CT scans did not reveal any fracture of the skull base. Upon examination, the patient regained consciousness. There were no significant motor or sensory deficits in the extremities. The patient presented with right ptosis, associating with conjunctival chemosis and periorbital edema. There was almost complete abducents and mild oculomotor palasy. The palpation of a zygomatic projection was suggestive of a linear fracture with no displacement. The nasal bones, mandible, and palate were intact. The jaw presented with an abnormal occlusion, and opened to 20 mm. Orthopantomography revealed left LeFort II and right LeFort III maxilla fractures, and inferior displacement of the bilateral zygomatic arches. Other radiographs of the periorbital area were not suggestive of a fracture. From the findings described above, a diagnosis of concomitant zygomatic and maxilla fracture was made. The findings of the right eye were striking. Pulsatile proptosis and vascular engorgement of the bulbar conjunctiva were marked. The upper and lower lids were edematic, and the upper lid was ptotic. Movement was limited, except downward and nasally, suggesting fairly good superior oblique function. The pupils reacted equally and consensually to light. Visual acuity was unchanged at 20/20 in both eyes. A 6-mm proptosis was noted. Funduscopy disclosed an outward venous engorgement and several small pre-retinal hemorrhages below the disc. Tonometry indicated a mild elevation of the intra-ocular pressure. The visual fields were normal. An ophthalmologic consultation considered superior orbital fissure syndrome.

**Figure 1:**
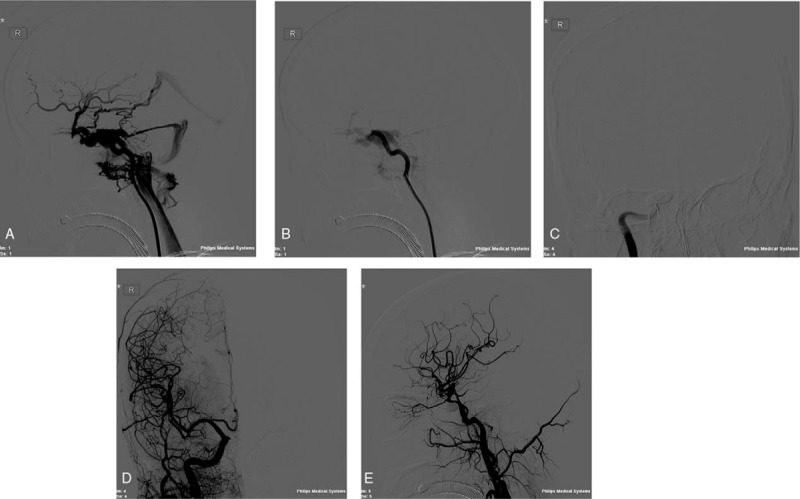
(A) A 32-year-old woman diagnosed with CCF secondary to a motor vehicle accident. DSA view of right internal c. A. revealed laceration in the horizontal segment and associated direct right CCF. (B) Preoperative right vertebral artery injections demonstrating direct CCF development. (C) Two coins embolization of the fistula were performed with preservation of the internal c. A. (D) Posttreatment frontal DSA view of right internal c. A. demonstrating obliteration of the fistula and lack of residual filling. (E) Posttreatment right common c. A. injection revealed lack of residual filling of the fistula. C. A. = carotid artery, CCF = cavernous sinus-carotid fistula, DSA = digital subtraction.

On the ninth day after admission, sudden bleeding from the mouth, nose, and trachea occurred, for which the patient received one unit of blood (400 mL), while right anterior and posterior nasal packs were inserted. The patient was transferred to the Intensive Care Unit. The bleeding gradually stopped. Thereafter, the patient began to notice pulsatile noises coming from the right side of her head, followed by proptosis and an increase in the intraocular pressure. The patient presented with diplopia. During the next few days, visual acuity in the right eye was gradually reduced to 20/70. The pounding, roaring sound, which associated with an orbital frontal headache, became intolerable to the patient. Digital subtraction angiography (DSA) was underwent. Bilateral selective trans-femoral carotid angiograms confirmed the presence of a discrete right CCF. There was no significant steal or crossover into the left cavernous sinus. Cross-compression films revealed good filling from the opposite carotid system (Fig. 1A, B). A surgical approach was devised in order to occlude the fine fistula utilizing a coin. During the preliminary occlusion of the right ICA with a balloon, the patient suddenly experienced left arm and leg palsy, and the surgery was terminated. The surgeon believed this due to an ICA spasm, and recommended mata's exercise. After a month, occlusion of the right traumatic CCF (TCCF) was performed. The cavernous sinus was reached through the right femoral artery by coaxial bouginage. Electrocephalography showed no abnormalities in the neurologic state of the patient during the surgery, and 2 coins were placed inside (Fig. 1C). The complete occlusion of the fistula was achieved, and the subjective or objective murmur disappeared. The carotid arteries continued to deliver a good blood supply into the intracranial circle that was previously excluded (Fig. 1D, E). Clinically, the pulsatile protosis, oculomotor palsy, and bruit immediately improved, whereas the abducens palsy and diplopia improved gradually. In addition, the restricted opening of the mouth recovered. Two months after the embolization, the subjective and objective ophthalmic symptoms were completely resolved, although right inferior hemianopsia persisted as a sequela of the CCF.

## 3 Discussion

### 3.1 Dissection

TCCF, a direct arteriovenous shunt, is caused by a tear in the intracavernous carotid artery (ICA) or one of its branches, leading to direct communication between the ICA and the cavernous sinus. The cavernous sinus, which localizes to the lateral part of the sphenoid bone, is a plexus surrounding the c. A in this specific area of the skull. The internal c. A. lies on the medial wall of the cavernous sinus. The cavernous sinus also contains cranial nerves III, IV, and VI, as well as the ophthalmic and maxillary divisions of the trigeminal nerves.^[3]^ These nerves pass forward through the cavernous sinus. The internal c. A. begins at the petrous apex after exiting from the carotid canal, and it ascends within the cavernous sinus slightly medial toward the posterior cavernous sinus and slightly medial toward the posterior clinoid process. It continues to run anteriorly along the carotid sulcus of the sphenoid bone, and then curves upward on the medial side of the anterior clinoid process. After it enters the cavernous sinus, it runs medially to cranial nerves III, IV, and VI, and laterally to the ophthalmic branch of cranial nerve V. The medial wall of the cavernous sinus is between the artery and pituitary gland. Finally, the internal c. A. penetrates the dura, running in posterior, medial, and superior directions.

There are 5 sites at which a fistula can occur. The incidence of a fistula at each of these sites, according to Debrun's data on 54 patients with CCF is as follows: 1 case (2%) on the anterior ascending segment, 5 cases (9%) at the junction of the anterior ascending and horizontal segment, 22 cases (41%) on the horizontal segment, 15 cases (28%) at the junction of the horizontal and posterior ascending segment, and 11 cases (20%) on the posterior ascending segment.^[4]^ In the case presented in this paper, the location of the fistula was on the horizontal segment. According to Nossek's classification, TCCFs can be divided into type I or type II.^[5]^ Type I is characterized by a single tear in the intracavernous c. A, thus forming a high flow arteriovenous fistula. Type II is characterized by broken branches of the intracavernous c. A. Furthermore, the anterior cavernous sinus communicates with the superior and inferior ophthalmic veins. Arterialization of this system leads to local venous hypertension and the previously described eye sings posterior communications with the petrosal sinuses and basal venous plexus overlying the clivus have clinical significance in this condition.

### 3.2 Etiological factors

#### 3.2.1 TCCF

Trauma is common to most medical conditions, and it follows orthognathic, plastic, and minor surgeries, such as phinoplasty,^[6]^ Ext.Ethmoidsurgery,^[7]^ Biopsy,^[8]^ LeFort I osteotomy.^[9]^ These cases are defined as traumatic, with males outnumbering females at a ratio of 2:1. They usually associate with a skull base, frontal or mid-facial fracture. For example, the intracavernous region of the arterial wall, which is surrounded by an irregular plexus of venous channels, can be torn or lacerated by a bony spicule or a shearing force (to which it is extremely susceptible, as it is fixed by the carotid canal posteriorly and by the dura anteriorly). Furthermore, its vasa vasorum may be damaged, thus producing a dissecting aneurysm, which will eventually be ruptured into the cavernous sinus ensues.^[1,10]^

#### 3.2.2 SCCF

The causes of SCCF are more difficult to ascertain. SCCF is more common in older women with increased incidence during pregnancy; however, it rarely occurs in males. SCCF can be due to a pre-existing intracavernous carotid aneurysm, defect in the carotid wall, or traumatic rupture. The most common cause of SCCF is a rupture of a pre-existing subclinical carotid aneurysm. In 12% to 15% of cases, carotid aneurysms occurred bilaterally, which is probably due to a residual remnant of the primitive trigeminal artery.^[11]^

#### 3.2.3 Clinical symptoms

The symptoms of CCF are generally monolateral, usually occurring at an average interval of 4 weeks after trauma, although this interval may range from a few hours to 1 year. Thus, the onset of symptoms may vary. The symptoms that are commonly associated with CCF include proptosis (94%), pulsating exophthalmos (40%), bruit (75%), orbital frontal headache and orbital pain (40%), chemosis (71%), extraocular palsy and diplopia (60%), loss of visual acuity (46%), and fifth nerve involvement (24%).^[12–14]^ The pressure gradient resulting from the carotid–cavernous communication creates a retrograde flow into the ophthalmic veins. As a result, several symptoms may be noted. Firstly, pulsating exophthalmos may be evident, which can be eliminated by compressing the homolateral common c. A. Pulsation with an orbital headache and pain may also be felt in the affected temporal and parietal areas, as well as subjective or objective bruit. Its intensity is often higher on the fistula side, particularly at the level of the involved orbit, and dilation of superficial veins of the eye lids and forehead, chemosis, pulsating retinal veins, papilloedema, loss of visual acuity due to impaired retinal circulation and possibly, late optic atrophy, may also be evident. Secondly, diplopia, the most frequent symptom, is secondary to compression of the oculomotor nerve in the cavernous sinus, which maintains a close relationship with the c. A., and its involvement will result in a limitation of lateral gaze. The IIIrd and IVth nerves may also be affected, resulting in opthalmoplegia. The ophthalmic and maxillary branches of the Vth nerve, when affected, will cause sensory disturbances, while the VIIth and VIIIth cranial nerves will become involved as the haematoma increases or the intraorbital pressure rises. Thirdly, ptosis is caused by the loss of sympathetic supply to Muller's muscle or the disruption of the innervation of the levator palpebrae. Fourthly, disruption of the parasympathetric innervation may result in a fixed, dilated pupil. The latter, which associates with ptosis and anhydrosis, is suggestive of Horner's syndrome.

#### 3.2.4 Different diagnoses

Other conditions with similar symptoms include superior orbital fissure syndrome, orbital apex syndrome, orbital (retrobulbar) hematoma, and cavernous sinus thrombosis. Cavernous sinus thrombosis can occur in the absence of trauma. The exophthalmos related to CCF usually progresses for several days and then stabilizes. With orbital hematoma, there is a tendency toward the spontaneous resolution of exophthalmos. The loss of visual acuity with orbital hematoma is not common. The presence of orbital bruit is diagnostic, and it is characteristic of only CCF. The symptom that is most distressing to patients is hearing a hum, swish, or roar, which can be effectively suppressed by carotid compression. A recent history of dental treatment, facial infection with an elevated white blood cell count, or leukocytosis can be suggestive of cavernous sinus thrombosis. Furthermore, superior orbital fissure syndrome and orbital apex syndrome occur most frequently in conjunction with LeFort II and III maxilla fractures, which can involve the optic foramen and superior orbital fissure, thus disrupting the sensory and motor innervation to the orbit and adjacent structures. Direct orbital trauma without evidence of fracture and with all the symptoms discussed, except infection, bruit, and pulsating exophthalmos, suggests orbital hematoma. Plain and tomographic films indicating fractures of the sphenoid complex should lead the physician to a high index of suspicion for CCF. The single most important diagnostic study should be cerebral angiography when diffusion of the radiopaque material into the cavernous sinus during the filling phase is noted. This indicates the presence of a fistula; it also measures its size, determines the flow to the distal supraclinoid vessels, and most importantly indicates the competency of the circle of Willis and contralateral carotid circulation.^[15,16]^

#### 3.2.5 Treatment

The treatment modalities for CCF involve dispelling the blood vessel in the cranium, allowing contraction of the projecting eye, rescuing eyesight, and preventing blood loss from the brain (i.e., hemorrhage). The treatment depends on the speed of blood flow through the fistula and its arterial supply, and the routes of venous drainage. The basic principle is to correct circulatory hemodynamics. However, there have been a few reports of spontaneous closure of such a fistula after accidental severe hypotensive episodes, and approximately 25% to 50% of dural CCFs will close spontaneously. The detachable balloon approach is considered by most neurosurgeons to be the best initial therapy.^[16]^ However, percutaneous balloon embolization is currently the procedure of choice, because it allows preservation of the ICA. A previous study has reported that the success rate of balloon embolization is 80%.^[16]^ Surgical techniques are generally indicated when the ICA has been previously ligated or the balloon cannot be inserted into the cavernous sinus. If the detachable balloon approach fails or the fistula and ICA have been occluded but the fistula reoccurs, then surgery is the last resort. Surgery involves ligation or occlusion of the common, internal, and external carotid arteries or embolization of the intra-cavernous portion of the internal c. A. Although complications can arise, the success rate is 50%.^[3]^ In this study, one female patient who underwent common arterial ligation presented with motor aphasia and facial paralysis. Satisfactory resolution often depends on the severity of the symptoms and the time interval between the onset and treatment of the condition.

## 4 Conclusions

The successful treatment of craniofacial injuries is critical during oral maxillofacial surgery. CCF is a rare complication arising from cranial or facial trauma. In general, few cases have been reported in the literature. For example, Yasnbaru examined 20 cases of traumatic or introgenic CCF in the oral and maxillofacial literature from 1970 to 1990.^[3]^ Because dentists and oral surgeons can detect the signs of CCF at early stages of trauma, they can provide an accurate diagnosis and execute correct treatment so as to achieve successful results. The reasons for clinical misdiagnosis are that CCF may appear at any time after injury and different symptoms may arise in different patients. Furthermore, head injuries may affect the final diagnosis. Mild chemosis and bruit are the initial symptoms of early CCF. Although CCF is outside the treatment specialization of the head and neck surgeon, he/she must be aware of all the ramifications of trauma in order to provide optimal patient care in case an irreversible disastrous complication occurs. In summary, we emphasize the need for a team-based approach to total patient care.
